# First reported *Porrocaecum angusticolle* infection in Griffon vulture (*Gyps fulvus*) in China

**DOI:** 10.3389/fcimb.2023.1181999

**Published:** 2023-07-11

**Authors:** Gongzhen Liu, Qing Liu, Wei Zhang, Xuewen Shen

**Affiliations:** ^1^ College of Agriculture and Forestry, Linyi University, Linyi, Shandong, China; ^2^ Animal Zoo Department, Jinan Park Development Service Center, Jinan, Shandong, China; ^3^ Honghe Hani and Yi Autonmous Prefecture Agriculture and Rural Affairs Bureau, Animal Disease Control Center, Mengzi, Yunnan, China

**Keywords:** *Porrocaecum angusticolle* (P. angusticolle), Griffon vulture (*Gyps fulvus*), diagnose, PCR, China

## Abstract

This present study is the first case of a *Porrocaecum angusticolle* (*P. angusticolle*) infection reported in *Griffon vulture* (*Gyps fulvus*) in China. This study aimed to identify the nematode species and explore the genetic evolution of worms infecting *Gyps fulvus* (*G.fulvus*). Clinical examination revealed several milky white parasites in the stomach and intestinal tract. Polymerase chain reaction and partial 18S gene sequencing analyses identified these worms to be *P. angusticolle* (SD isolates). Further phylogenetic analyses revealed that they shared the highest genetic identity (99.9%) with a *P. angusticolle* isolate (EU004820.1) from Germany. Our study is the first report on the identification and characterization of *P. angusticolle* infecting *G.fulvus* in China, based on clinical findings and molecular diagnosis. Therefore, our study provides novel insights for the diagnosis of *P. angusticolle* infections and the prevention of nematode transmission in wild and domestic animals.

## 1 Introduction

*Porrocaecum angusticolle* (*P. angusticolle*) is among the pathogenic nematodes infecting various birds (Mozgovoi, 1953; Digiani and Sutton, 2001; Li et al., 2015) and occasionally mammals (Sprent, 1973; Jian, 1989). To date, approximately 40 *Porrocaecum* nematode species have been reported. Briefly, *P. angusticolle* is classified into Eukaryota, Metazoa, Nematoda, Choromadorea, Rhabditida, Ascarididae, and *Porrocaecum.* Previous studies have reported *P. angusticolle* infections in Europe, mainly in Italy (Santoro et al., 2010), Portugal (Tomás et al., 2017), the Czech Republic (Kijewska et al., 2002; Guo et al., 2021), Germany (Honisch and Krone, 2008), and Spain (Sanmartín et al., 2004; Santoro et al., 2012). Several birds species have been reported to be infected by *P. angusticolle*, including *Buteo buteo* (Kijewska et al., 2002; Santoro et al., 2010; Guo et al., 2021), *Strigiformes*, *Aquila clanga*, *Aquila chrysaetos*, *Accipiter gentilis*, *Accipiter nisus*, *Aquila pomarine*, *Aquila rapax*, *Accipiter striatus*, *Buteo jamaicensis*, *Buteo lagopus*, *Buteo platypterus*, *Circus aeruginosus*, *Circus cyaneus*, *Circaetus gallicus*, *Elanius caeruleus*, *Haliaeetus albicilla*, *Haliastur indus*, *Milvus milvus*, *Milvus migrans*, *Pernis apivorus*, *Pandion haliaetus* (Santoro et al., 2012), *Sparrowhawk* (Min et al., 2021), *Circus aeruginosus* (Kijewska et al., 2002), *Accipiter gentilis*, *Accipiter nisus*, *Buteo lagopus*, *Falco subbuteo*, *Milvus migrans*, *Pandion haliaetus* (Honisch and Krone, 2008), *Tyto alba*, and *Strix aluco* (Sanmartín et al., 2004). However, limited information exists on *P. angusticolle* infections in the literature.

Notably, a *P. angusticolle* infection is typically diagnosed based on clinical symptoms, such as pathological lesions, and molecular diagnosis. To the best of our knowledge, only 16 nucleotide sequences of the *P. angusticolle* genome have been submitted to the GenBank database, including those of the 18S, 28S, COX, and ITS genes. In the present study, we used primers targeting the 18S gene to perform sequencing of avian samples for the diagnosis of infection and identification of nematode worms. Interestingly, by combining the evaluation of clinical symptoms and molecular identification, we diagnosed, for the first time, a case of *P. angusticolle* infection of *G.fulvus* in China.

## 2 Methods

### 2.1 Case presentation

In January 2022, we were notified that a male *G.fulvus* older than 10 years of age had died. Following dissection, we found dozens of milky white parasites in its stomach and intestinal tract, with the size of worms ranging from 7 to 15 cm. We then collected these parasitic worms from the deceased vulture for further studies. We also examined the bird and observed clinical signs of the intestinal tract showing hemorrhagic spots and anabrosis.

### 2.2 Polymerase chain reaction analysis

We washed each parasite with double distilled water three times for 5 min each time. Subsequently, we added 20 μL of proteinase K (Vazymy, Nanjing, China) in each worm sample, vortexed for mixed the sample, and incubated it at 56°C (Constant temperature) for at least 3 h. We then subjected the digested parasite suspension to DNA extraction using the FastPure Cell/Tissue DNA Isolation Mini Kit (Vazymy). Each PCR amplification system included 12.5 µL Taq DNA Polymerase Mix (Transgen Co., Beijing, China), 10.5 µL ddH_2_O, forward and reverse primers (100 pmol/µL, 0.5 µL each), and 1 µL template DNA in a total volume of 25 µL. We also used double distilled water instead of template DNA as a blank control. We used 3 pairs of primers (NC1-NC3) designed in previous studies to amplify the small subunit DNA segment (18S), cytochrome oxidase I (COX1), and internal transcribed spacer (ITS) genes (Folmer et al., 1994; Gasser et al., 1999; Floyd et al., 2005). Three primer pairs followed as: 18S(NC1F), 5′-CGCGAATAGCTCATTACAACAGC-3′ and 18S(NC1R), 5′-GGGCGGTATCTGATC GCC-3′; COX1(NC2F), 5′-GTAGGTGAACCTGCGG AAGGATCATT-3′ and COX1(NC2R), 5′-TTAGTTTCTTTTCCTCCGCT-3′; ITS(NC3F), 5′-GGTCAACAAATCATAAAGATATTGG-3′ and ITS(NC3R), 5′-TAAACTTCAGGGTGAC CAAAAAATCA-3′. The PCR amplification conditions were as follows: pre-denaturation at 95°C for 5 min; 35 cycles of denaturation at 95°C for 30 s, annealing at 56°C for 30 s, and extension at 72°C for 60 s; and a final extension at 72°C for 10 min.

### 2.3 Gene sequence and analysis

Following recovery and purification using the Agarose Gel DNA Extraction Kit, PCR products were sequenced by Biosune Biotechnology Co., Ltd. We analyzed the obtained sequencing results using the MEGA and DNAstar software and compared the identified sequences of 18S, COX1, and ITS genes of *P. angusticolle* with those of other nematodes. First, we used the GenBank and PubMed online websites to perform a comparative analysis of the sequences of highly homologous geographical parasite strains, especially their 18S sequences. In addition, using the DNAstar MegAlign Pro with the Clustral W algorithm, we analyzed the distance and divergence of these sequences. Subsequently, we used the Kimura 2-parameter model and maximum likelihood method in MEGA 6.0 software to draw the phylogenetic tree.

## 3 Results and discussion

**Figure 1:**
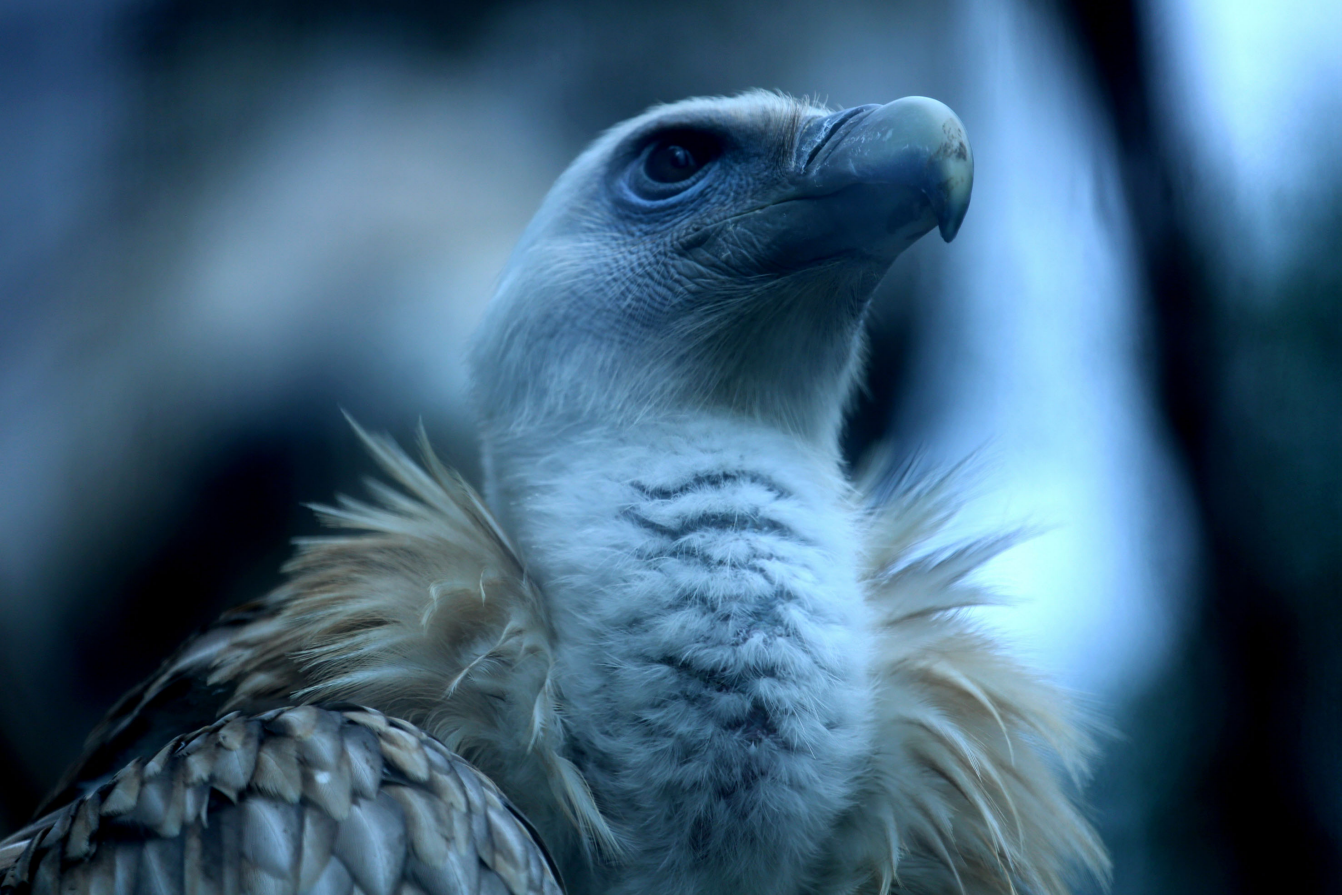
Image of a *G.fulvus*. This is partial head photograph of *G.fulvus* in animal zoo.

**Figure 2:**
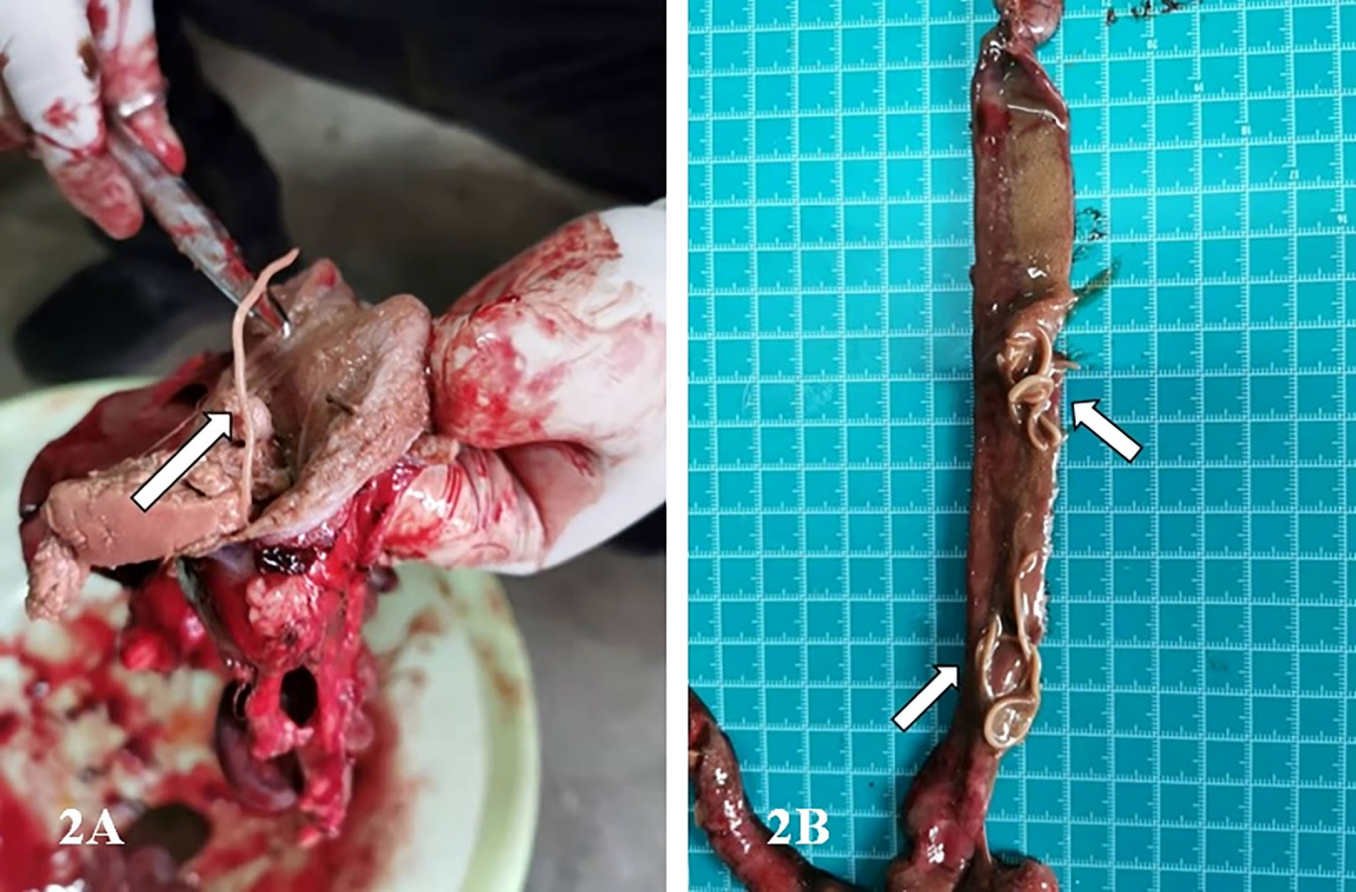
Images showing the infected stomach and intestinal tract of the *G.fulvus* bird. **(A)** One milky white parasite in the stomach of *G.fulvus*; **(B)** Dozens of milky white worms in the intestinal tract of *G.fulvus*.

Decreased immune function following parasite infection might be one of the potential risk factors leading to the death of *G.fulvus* birds (**Figure 1**) (Guivier et al., 2017; Lima and Lodoen, 2019). In the present study, we report the first case of an identified *P. angusticolle* infection of *G.fulvus*, confirmed by clinical findings and molecular diagnosis (Cabezón et al., 2011; Darwich et al., 2012; Chakarov and Blanco, 2021). Notably, we identified adult worms in the stomach and intestinal tract of the bird instead of nematode eggs (**Figure 2**). Previous studies have reported the occurrence of approximately 40 nematode species worldwide (Li and Scholz, 2019), including *Porrocaecum semiteres* (Syrota and Kharchenko, 2015), *Porrocaecum ensicaudatum* (Kijewska et al., 2002), *Porrocaecum aridae*, *Porrocaecum crissum deslong*, *Porrocaecum praelongum*, *Porrocaecum reticulatum* (Li et al., 2015), *Contracaecum multipapillatum* (Navone et al., 2000; Valles-Vega et al., 2017), *Contracaecum micropapillatum*, *Contracaecum bancrofti*, *Contracaecum variegatum*, *Contracaecum eudyptulae*, and *Contracaecum ogmorhini* (Shamsi et al., 2009). To date, the complete lifecycle of most *Porrocaecum* and *Contracaecum* species remains unclear. Only a few species have been reported to include intermediate hosts, such as earthworms (Moravec, 1971), insectivores (Portolés et al., 2004), and fish (Moravec, 2009). Under suitable environmental temperatures at the range of 22–32°C, eggs are hatched into active larvae, which enter the earthworm, developing into invasive larvae and cysts after 2 months. Following feeding of the terminal host on an infected worm-containing carcass, the invasive larvae in the worm develop into adults in the small intestine of the terminal host over 3 weeks. For example, in the case of *C. multipapillatum*, the first intermediate host is the cyclops, while the second intermediate host is fish (Moravec, 2009).

Previous studies have mainly focused on the morphological and genetic characterization of cases of *P. angusticolle* infections in *B. buteo*, *Strigiformes*, *A. clanga*, *A. chrysaetos*, and other birds (Kijewska et al., 2002; Santoro et al., 2010; Santoro et al., 2012; Tomás et al., 2017; Guo et al., 2021). Therefore, information on the lifecycle of *P. angusticolle* is still lacking. In these previous studies, adult *P. angusticolle* worms were typically found in the superficial layer of the stomach and intestinal tract. Interestingly, both *Porrocaecum* larvae and adult worms can drill into the mucous membrane of the gastric wall of the glandular stomach to produce hemorrhagic spots, bruises, and erosive ulcers, which affect the growth and development of birds, and can lead to death in cases of severe infection (Mozgovoi, 1953; Guo et al., 2021).

**Figure 3:**
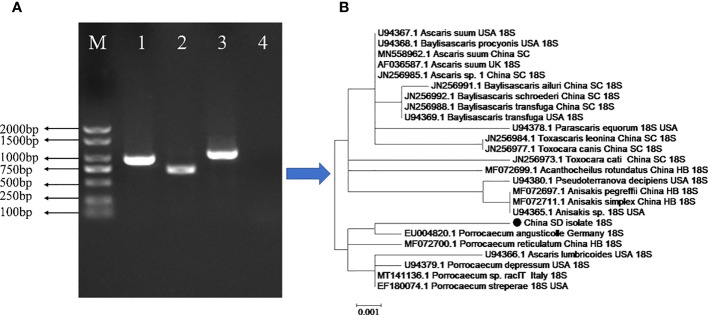
Genetic characterization of the parasite infecting the stomach and intestinal tract of the *G.fulvus* bird. **(A)** PCR amplification of the 18S, COX1, and ITS genes of *P. angusticolle.* M: Marker; Lane 1:18S gene; Lane 2: COX1 gene; Lane3: ITS gene; Lane 4: Blank control. **(B)** Phylogenetic tree construction of the *P. angusticolle* 18S gene with high homology of 24 geographical nematode isolates.

In the present study, genetic analyses revealed that the partial sequence of the identified 18S rRNA fragment of *P. angusticolle* in our study (**Figure 2**) exhibited the highest phylogenetic identity (99.9%) with that of an isolate from Germany (Germany: EU004820.1). Subsequently, we analyzed the phylogenetic relations of our *P. angusticolle* strain to that of other *Porrocaecum* species, such as *P. reticulatum* (China HB: MF072700.1), *P. depressum* (USA: U94379.1), *Porrocaecum* sp. (Italy: MT141136.1), and *P. streperae* (USA: EF180074.1), and found that they ranged from 99.6 to 99.9% (**Figure 3**). The generated phylogenetic tree further confirmed the evolutionary relationship between *Porrocaecum* and other nematode species (**Figure 3**). In conclusion, in our study, we performed 18S gene analysis to identify and characterize a *P. angusticolle* infection in *G.fulvus* in China. We also examined the COX and ITS genes; however, limited information on the phylogenetic relationships of these genes is available in PubMed and GenBank.

The *G.fulvus* is a bird species belonging to the Accipitridae family of vultures. It inhabits a wide range of habitats, reaching up to 2500 m above sea level, such as rocky alpine and plateau areas, grasslands, and scrub and semi-desert areas (Davidović et al., 2020; Pirastru et al., 2021). Owing to its excellent night vision, *G.fulvus* forages for dead animals during the night while feeding on wild animals such as goats, deer, and gazelles in the daytime, relying on its sensitive smell to locate decaying animal carcasses (Marin et al., 2014; Sevilla et al., 2020). It is widely distributed throughout Europe, the Middle East, and North Africa, as well as in India and the Himalayas. However, it is most common in countries bordering the Mediterranean Sea, with the largest population number detected in Spain, accounting for three-quarters of the European population (Davidović et al., 2022). To the best of our knowledge, this study is the first report on the identification and genetic characterization of *P. angusticolle* infection in *G.fulvus* in China.

Although this study is the first report of *P. angusticolle* infection in *G.fulvus*, it has some limitations. First, although we combined PCR methods, gene sequencing, and clinical factors to diagnose the *P. angusticolle* infection, there are no completely set diagnostic criteria for *P. angusticolle*. In addition, we did not record enough clinical pictures and symptoms to support our diagnosis, and few studies have reported *P. angusticolle* infection in birds.

Nevertheless, our study extends the current geographical distribution and host species of *P. angusticolle*, confirming the spread and genetic evolution of this nematode in Asia and highlighting the importance of the molecular diagnosis of *P. angusticolle* infections in domestic and wild animals.

## Data availability statement

The Genebank accession ID: OQ216840,which can be found below: https://www.ncbi.nlm.nih.gov/genbank/.

## Ethics statement

Written informed consent was obtained from the participant/patient(s) for the publication of this Brief Research Report.

## Author contributions

GL designed the study and drafted the manuscript. QL, WZ and XS collected the animal specimens and supported the experiment. All persons who have made substantial contributions to the work are reported in the manuscript. All authors contributed to the article and approved the submitted version.
